# Mother-to-Child Transmission of Human T-Cell Leukemia Virus Type 1: Mechanisms and Nutritional Strategies for Prevention

**DOI:** 10.3390/cancers13164100

**Published:** 2021-08-14

**Authors:** Kazuo Itabashi, Tokuo Miyazawa

**Affiliations:** 1 Aiseikai Memorial Ibaraki Welfare Medical Center, 1872-1 Motoyoshida-cho, Mito-City 310-0836, Japan; 2 Department of Pediatrics, Showa University School of Medicine, 1-5-8 Hatanodai, Shinagawa-ku, Tokyo 142-8666, Japan; miyazawa.t@med.showa-u.ac.jp

**Keywords:** human T cell leukemia virus type 1, mother-to-child transmission, exclusive formula feeding, short-term breastfeeding, frozen and thawed breastmilk feeding, transplacental transmission

## Abstract

**Simple Summary:**

Mother-to-child transmission (MTCT) of human T-cell leukemia virus type 1 (HTLV-1) is a major cause of adult T-cell leukemia (ATL). Owing to the poor prognosis of ATL and the fact that more than one million people have been infected with this virus, the HTLV-1 antibody screening test was established in Japan in 2010 for all pregnant women to detect carriers and prevent MTCT. Because breastfeeding is the most common route of postnatal MTCT, exclusive formula feeding is widely used as a measure to prevent MTCT. Recently, it was reported that there is no obvious difference in the efficacy of short-term breastfeeding for ≤3 months in preventing MTCT compared to that in exclusive formula feeding, and that a duration of breastfeeding that does not exceed four months can be effective for preventing MTCT.

**Abstract:**

Approximately 95% of mother-to-child transmission (MTCT) of human T-cell leukemia virus type-1 (HTLV-1) is derived from prolonged breastfeeding, which is a major cause of adult T-cell leukemia (ATL). Exclusive formula feeding (ExFF) is therefore generally used to prevent MTCT. A recent cohort study revealed that 55% of pregnant carriers chose short-term breastfeeding for ≤3 months in Japan. Our meta-analysis showed that there was no significant increase in the risk of MTCT when breastfeeding was carried out for ≤3 months compared with ExFF (pooled relative risk (RR), 0.72; 95% confidence interval (CI), 0.30–1.77), but there was an almost threefold increase in risk when breastfeeding was carried out for up to 6 months (pooled RR, 2.91; 95% CI, 1.69–5.03). Thus, short-term breastfeeding for ≤3 months may be useful in preventing MTCT. Breastmilk is the best nutritional source for infants, and any approach to minimizing MTCT by avoiding or limiting breastfeeding must be balanced against the impact on the child’s health and mother–child bonding. To minimize the need for nutritional interventions, it is necessary to identify factors that predispose children born to carrier mothers to MTCT and thereby predict MTCT development with a high degree of accuracy.

## 1. Introduction

Early-life exposure to infectious agents may be involved in the development of future cancers. Well-known pathogens include the human papillomavirus, hepatitis B and C viruses, Epstein–Barr virus, and human T-cell leukemia/lymphoma virus type-1 (HTLV-1) [1]. Among these pathogens, the number of HTLV-1 carriers in Japan is by far the largest among developed countries, estimated to be at least 1.1 million based on data from first-time blood donors in 2006 and 2007 [2]. While the majority of HTLV-1-infected individuals remain asymptomatic, it is well known that adult T-cell leukemia/lymphoma (ATL) and HTLV-1-associated myelopathy/tropical spastic paraparesis (HAM/TSP) are caused by this virus. HTLV-1 carriers are estimated to have a lifetime risk of 2–7% for the development of ATL [3] and 0.25–3.8% for the development of HAM/TSP [4]. The pathogenesis of HAM/TSP and other HTLV-1-associated diseases, such as infective dermatitis and myositis, are derived from inflammation due to HTLV-1 infection [4,5]. HTLV-1 uveitis, which has long been recognized in the field of ophthalmology, is also associated with inflammation caused by HTLV-1 [6]. Infective dermatitis associated with HTLV-1 (IDH) is a recurrent eczema that affects vertically infected children [7,8]. Although IDH disappears in adulthood, it may predispose individuals to the early development of HAM/TSP and ATL [9,10].

A recent meta-analysis demonstrated that the risk of all-cause death was higher in people with HTLV-1 infection than that in people without the infection [11]. The analysis indicated that many of the diseases associated with HTLV-1 are not fatal, and those that are fatal (e.g., ATL) occur too rarely to account for the observed mortality effect. Thus, HTLV-1 infection is likely to affect human health in more ways than is currently unknown, and with increasing globalization, it has the potential to spread from endemic to non-endemic areas and become a global burden.

Diverse clinical features, including lymphadenopathy, skin lesions, increased abnormal lymphocytes, frequent blood and bone marrow involvement, hypercalcemia, and lytic bone lesions characterize ATL [12]. The diagnosis of ATL often involves the detection of ATL cells (‘flower cells’) in the peripheral blood. ATL has been divided into four clinical subtypes based on the Shimoyama classification system: acute, lymphoma, smoldering, and chronic [13]. The smoldering and chronic subtypes, also known as indolent ATL, are characterized by rashes and minimal blood involvement. The acute and lymphoma subtypes, also known as aggressive ATL, are characterized by a large tumor burden, lymph node and blood involvement, and hypercalcemia. Classification of ATL subtypes greatly influences the treatment regimen and prognosis of patients [14].

ATL has been associated with HTLV-1 mother-to-child transmission (MTCT) [1] owing to the following reasons: (1) ATL develops after a long incubation period of more than 20–30 years [3]; (2) the majority of ATL patients are infected during childhood [15]; (3) the development of ATL is extremely rare in people infected in adulthood [3]; (4) breast milk containing infected cells is the main route of transmission during this period [16,17,18,19]; and (5) family history is a risk factor for developing ATL [20]. Numerous studies have demonstrated that MTCT through breastfeeding is the predominant route of HTLV-1 infection [15,16,21], and HAM/TSP develops in populations infected through vertical and horizontal routes [22]. Recently, a case of HTLV-1-associated uveitis caused by horizontal transmission was reported [23].

In Japan, there has been a nationwide antenatal HTLV-1 antibody screening program since 2010 to prevent HTLV-1 MTCT [24,25]. Because infected children are often asymptomatic during childhood, it is not clear whether MTCT is involved in the development of HTLV-1-associated diseases other than ATL. It is expected that an antenatal HTLV-1 screening program will reduce the number of infected children via MTCT, which in turn will reduce the number of ATL cases. Furthermore, the reduction in the number of these children may also contribute to a reduction in the sources of horizontal transmission. The following are the justifications for nationwide screening in Japan: (1) HTLV-1 carriers are widespread throughout Japan because of internal population migration from endemic areas such as Kyusyu to non-endemic areas [2]; (2) more than 4000 adolescents and adults (77% women) are newly diagnosed annually with HTLV-1 (mainly caused by sexual contact) [26]; and (3) no effective vaccine or antiviral regimens have been developed against this virus [27].

However, despite the implementation of nationwide antenatal HTLV-1 antibody screening, there is no consensus among healthcare providers, especially regarding prevention by nutritional regimens. In this review, the mechanisms of MTCT and evidence for preventive measures through nutrition will be discussed along with the latest findings.

## 2. Nationwide Antenatal HTLV-1 Antibody Screening

### 2.1. International Trends in the Implementation of Nationwide Antenatal Screening

The implementation of nationwide HTLV-1 antibody screening tests in all pregnant women is controversial. Although the United Kingdom National Screening Committee considered the antenatal HTLV-1 screening program three times, the committee did not recommend introducing a screening program in the United Kingdom because of the low prevalence of HTLV-1 infection and the low risk for infected infants developing serious illness [28]. Although antenatal HTLV-1 screening is performed in some Brazilian cities and states, such as Salvador, the city with the highest reported HTLV-1 prevalence, it is not included among the tests currently offered to pregnant women by the Brazilian health system [29]. Nevertheless, it has been emphasized by several groups that screening tests for pregnant women are necessary in endemic areas and countries [29,30,31]. However, Japan is the only country to have a nationwide screening program for pregnant women.

### 2.2. Screening Program in Japan

**Figure 1:**
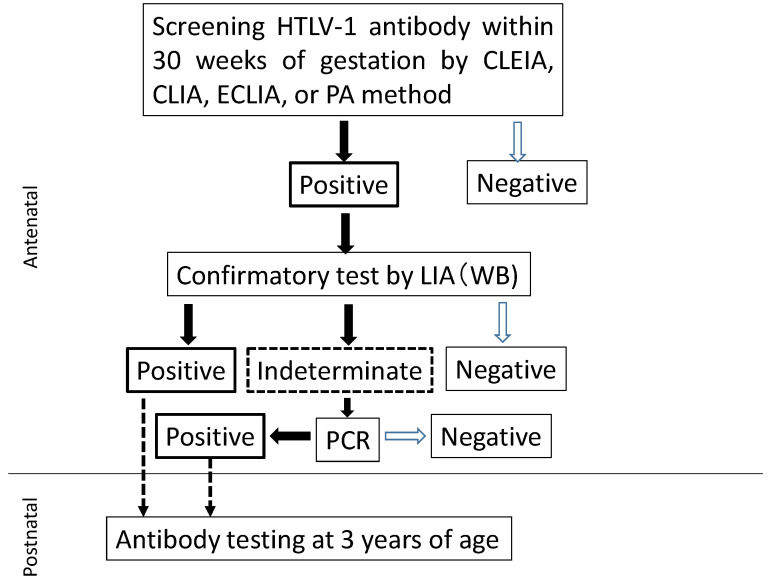
Flowchart to determine HTLV-1 carriers among pregnant women. CLEIA, chemiluminescent enzyme immunoassay; CLIA, chemiluminescent immunoassay; ECLIA, electro-chemiluminescent immunoassay; PA, particle agglutination; WB, Western blot; LIA, line immunoassay; PCR, polymerase chain reaction.

The flowchart for HTLV-1 carrier screening during pregnancy in Japan is shown in Figure 1. HTLV-1 antibody screening is usually performed within the first 30 weeks of gestation to secure enough time for a carrier to receive detailed information from healthcare providers and to select a suitable feeding regimen for their infants before labor. Confirmatory tests are performed on pregnant women with positive screening results. Line immunoassay (LIA) has demonstrated superior performance to that of Western blotting (WB), resulting in fewer indeterminate results [32,33]. Thus, WB has recently been replaced by LIA in Japan [33]. If the result of the confirmation test is indeterminate, a polymerase chain reaction (PCR) test is used to determine the presence or absence of infection. As shown in Table 1, pregnant women who had either a positive confirmatory test or PCR-positive results were identified as HTLV-1 carriers [25]. When a pregnant woman is identified as a carrier, the healthcare provider explains the risk of MTCT and preventive measures as much as possible before delivery. Our retrospective surveys conducted in 2011, 2013, and 2016 confirmed that a nationwide screening program for HTLV-1 was almost fully implemented in Japan [34]. Even if a child is born to a carrier pregnant woman, a regular infant’s health check-up schedule is appropriate unless the mother is highly anxious. Testing for HTLV-1 antibody at the age of 3 years to assess MTCT is recommended but not mandatory [25].

### 2.3. Prevalence among Pregnant Women in Japan

The prevalence of HTLV-1 carriers among pregnant Japanese women in 2011 and 2013 was 0.15% and 0.18%, respectively [35]. In 2019, the prevalence determined using the LIA and PCR was 0.10%. Among them, 10.7% had negative test results in their previous pregnancies, and the infections were therefore assumed to be due to horizontal transmission [36]. In Kyushu and Okinawa, which are endemic areas in Japan, the prevalence was 0.60%, 0.66%, and 0.30% in 2011, 2013, and 2019, respectively. The prevalence of HTLV-1 in pregnant women in 2019, both nationally and in the Kyushu and Okinawa areas, was lower than that in 2011 and 2013, but the reasons for this are not fully understood.

### 2.4. Worldwide Prevalence of HTLV-1 in Pregnant Women

Based on available data in 2012, Gessain and Cassar reported that the most endemic regions for HTLV-1 are the Southwestern part of Japan, sub-Saharan Africa and South America, the Caribbean region, and foci in the Middle East and Australo-Melanesia. They also reported the prevalence of HTLV-1 in pregnant women in these regions [37]. Rosadas and Tayler added to the published data regarding the prevalence of HTLV-1 infection among pregnant women after the report by Gessain et al. [18]. In South America, the prevalence among pregnant women was reported to be 0.1–1% in Brazil, 4% in French Guyana, and 1–4% in Peru. In the Caribbean area, the prevalence ranged from 2–4%. In sub-Saharan Africa, the prevalence was above 1% in all countries, and in Gabon, it was 5% in some areas [38]. As already mentioned, in Eastern Asia, Japan had a prevalence of 0.1% in 2019. In Europe, the prevalence in most countries was less than 0.1% (see Rosadas et al. for details on country data [18]).

## 3. Mechanisms of HTLV-1 MTCT

### 3.1. Cell-to-Cell Transmission

Human immunodeficiency virus (HIV), mouse mammary tumor virus (MMTV), and HTLV-1 are transmitted from mother to child through breast milk. HIV MTCT can occur before, during, and after delivery, with postnatal transmission through breastfeeding accounting for one-third to one-half of all cases of HIV MTCT [39]. Both cell-free and cell-associated viruses are present in the breast milk of HIV-infected mothers [40]. Ndirangu et al. reported that the role of cell-free viruses is more dominant than that of cell-associated viruses in MTCT through breast milk during the early postnatal period (6 weeks of life) [41]. MMTV is usually transmitted via breastmilk to the offspring, and neonatally infected mice of susceptible strains usually develop mammary tumors after only 5 months of life [42]. The MMTV-infected mother’s breastmilk contains cell-free viruses.

In contrast to HIV and MMTV, HTLV-1 cell-free viruses are rarely detected extracellularly [43,44]. Cell-free viruses are thought to be less involved in the spread of HTLV-1 infection. Thus, the spread of HTLV-1 infection is thought to occur predominantly through direct cell-to-cell contact. Subsequent experimental studies have shown that when dendritic cells (DCs) are exposed to cell-free viruses, the infection spreads to CD4+ T cells, but they may not be the main players for the spread of the infection [44,45,46,47].

In vitro studies have speculated that HTLV-1 cell-to-cell infection may spread through viral synapses [48], conduits [49], biofilm-like structures [50], and extracellular vesicles [51]. These modes allow the virus to escape elimination by the immune system (HTLV-1-specific T cell unresponsiveness) and efficiently deliver virions to contacted cells, resulting in an increased proviral load (PVL) [44,52,53]. HTLV-1 preferentially infects CD4^+^ T cells via their cellular receptors such as heparin sulfate (HS) proteoglycans and neuropilin 1 (NRP-1), which are used for the initial binding to the cell, and glucose transporter 1 (GLUT1) for entry [44,45,47,54,55]. However, Tanaka et al. found that the cellular susceptibility to HTLV-1 infection did not correlate with the expression of GLUT1, HS, or NRP-1 alone [56]. Cell-to-cell transmission of HTLV-1 can occur frequently after interactions between DCs and T cells, as well as between T cells [46,57]. Because DCs, monocytes, macrophages, and B cells express these receptors, they can also be infected with each other in individuals with HTLV-1 [44,58].

### 3.2. Modes of HTLV-1 Transmission

There are two modes of HTLV-1 transmission: horizontal infection due to sexual intercourse and blood transfusion, and antenatal or postnatal MTCT [59]. The most common mode in Japan is horizontal infection, with a prevalence of more than 4000 people infected per year [26]. The predominant horizontal infection is estimated to be related to sexual intercourse because donor screening for HTLV-1 infection is always tested at the time of blood donation [60]. Organ transplantation has also been identified as a mode of horizontal transmission of HTLV-1 [61]. Screening for HTLV-1 infection in donors of organ transplantation is recommended. Additionally, it is necessary to test whether the recipient is a carrier because the use of immunosuppressants may increase the risk of developing HTLV-1-associated diseases [62,63,64].

### 3.3. Routes of MTCT in HTLV-1 Infection

Currently, HTLV-1 MTCT is mainly attributed to prolonged breastfeeding based on the findings of epidemiological [65,66,67,68] and animal studies [69,70]. The ATL Prevention Program in Nagasaki revealed a marked reduction in HTLV-1 MTCT from 20.3% to 2.5% through exclusive formula feeding (ExFF) [16]. Previous studies revealed that the rate of HTLV-1 MTCT in children who were exclusively fed infant formula was significantly lower than that in children who were breastfed over a prolonged period. However, MTCT was observed in a small proportion of children who were exclusively fed infant formula [18,25]. This suggests the possibility of MTCT through antenatal routes. However, no evidence has been established for ascending HTLV-1 infection in utero, birth canal infection due to contaminated maternal blood exposure, or transplacental transmission. It is thought that more than 95% of MTCT cases are derived from prolonged breastfeeding, but even if antenatal routes constitute a small proportion of MTCT cases, it is necessary to elucidate the alternative infection route to prevent MTCT.

#### 3.3.1. Transplacental HTLV-1 Transmission

In in vitro experiments, exposure of the cell-free HTLV-1 virus to trophoblasts did not result in infection [71]. Recently, Tezuka et al. demonstrated that during pregnancy of HTLV-1 carriers, HTLV-1 was highly expressed in placental villous tissues, and villous trophoblasts showed high HTLV-1 sensitivity compared to that in other component cells (mesenchymal fibroblasts and placental vascular endothelial cells) of the blood–placental barrier. These results suggest that MTCT of HTLV-1 occurs through the placenta when the blood–placental barrier is impaired [72] (e.g., in preeclampsia [73]). However, the study could not directly investigate transplacental transmission because the authors did not have data on MTCT rates in children born to carrier pregnant women. Nevertheless, the study brings us one step closer towards understanding antenatal HTLV-1 MTCT.

#### 3.3.2. Transmission through Breastfeeding

It is not fully understood how HTLV-1-infected cells in breastmilk enter the infant and establish MTCT. Virus uptake during breastfeeding may occur in the tonsil mucosa or intestinal mucosa or in both of these sites in infants [74]. Although a recent in vitro study suggested that co-infection with HIV and cytomegalovirus can disrupt the mucosal barrier and allow HIV to spread to the tonsils [75], it is not clear whether it is involved as a transit pathway for HTLV-1.

Currently, postnatal infection in children born to carrier pregnant women is thought to occur primarily when infected cells in ingested breastmilk enter the infant’s digestive tract [70,76]. Animal studies have shown that breastmilk leucocytes survive passage through the infant’s digestive tract, and then translocate from the gastrointestinal tract to the blood and distant sites such as the lymph nodes, spleen, and liver [77,78]. The leukocyte count in breastmilk is highest in the colostrum and decreases to 0–2% of the total cell count within several weeks of lactation [79]. However, the rapid response of breastmilk leukocytes to infections of the mother and infant in healthy mother/infant dyads involves a tightly regulated process aimed at conferring additional immunological support to the infant [80]. Furthermore, there are many types of breastmilk cells other than leukocytes, including mammary luminal epithelial cells, lactocytes, and stem/progenitor breastmilk cells, whose relative proportions can change depending on the lactation period, maternal conditions, and infant feeding [79].

It was initially thought that HTLV-1 MTCT is mainly caused by CD4^+^ T cells [69,70], but the involvement of macrophages and mammary epithelial cells has also been considered because CD4^+^ T cells are not predominant in breastmilk. Southern et al. reported that basal mammary epithelial cells were susceptible to HTLV-1 infection and capable of transferring HTLV-1 infection to normal peripheral blood lymphocytes in an in vitro experiment [81,82]. Takeuchi et al. showed that breastmilk macrophages might be an appropriate HTLV-I reservoir involved in MTCT through breastfeeding [83]. These studies suggest that mammary epithelial cells and macrophages may be involved mainly in the persistence and transmission of HTLV-1 infection from carrier mothers. At present, it is not clear which cells present in breastmilk are the main players in transmission through breastmilk. It is necessary to longitudinally investigate the types and numbers of infected breast milk cells secreted by the carrier mother in the future.

The process from the contact of infected cells with the mucosa to the spread of infection in submucosal tissues has been described in detail in several reviews [44,84,85]. However, the process by which infected cells in breastmilk enter the infant’s digestive tract and establish infection has not been fully elucidated. After the HTLV-1-infected cells enter the digestive tract, infection likely involves the transfer of HTLV-1-infected cells and/or cell-free HTLV-1 produced by infected cells across the epithelium in the oral or gastrointestinal mucosa. In their review, Carpenter et al. summarized the process of establishment of HTLV-1 transfection after contact between mucosa and infected cells [84]. This could occur in the following ways: (1) the transit of a virion incorporated into a vesicle from the apical to the basal surface of an epithelial cell (transcytosis) [74]; (2) release of newly produced virions from the basal surface of an infected epithelial cell [86]; (3) bypass of HTLV-1-infected cells through a damaged mucosa [87]; and (4) transmigration of macrophages through an intact epithelium, as observed for HIV [88]. However, this has not yet been formally demonstrated [84].

## 4. Strategies to Prevent MTCT of HTLV-1

### 4.1. Prevention of MTCT by Measures Other Than by Nutrition

The strategies to prevent MTCT ideally involve the administration of neutralizing antibodies, vaccines, and antiviral drugs. An early study indicated the importance of conformational epitopes within HTLV-1 gp46 in mediating a neutralizing antibody response to HTLV-1 infection [89]. Fujii et al. evaluated the effects of passive immunization using an anti-gp46 neutralizing monoclonal antibody (LAT-27) in mice as part of their research to develop a vaccine. They found that neonatal mice born to mother mice pre-infused with LAT-27 showed complete resistance to intraperitoneal infection with HTLV-I. [90] However, if breastfeeding is continued after the period in which the antibody transferred to the newborn decreases or disappears, it is questionable whether it is effective in preventing mother-to-child infection. Therefore, an active vaccine that can elicit or boost anti-HTLV-I gp46 neutralizing antibody titers should be developed.

### 4.2. Prevention of MTCT by Nutritional Regimens

Shortly after the discovery of HTLV-1 approximately 40 years ago [91,92], it became clear that breastfeeding was the main route of MTCT. Therefore, prevention strategies have focused on either refraining from breastfeeding or reducing the infectivity of the carrier mother’s breastmilk. To date, the main nutritional strategy for the prevention of HTLV-1 MTCT is through ExFF [18,93]. In addition to ExFF, either short-term breastfeeding (STBF) or frozen and thawed breastmilk feeding (FTBMF) has been proposed in Japan [25,94].

The duration of breastfeeding is an important risk factor for MTCT and PVL in carrier pregnant women [31]. However, it is not clear after how many months of breastfeeding the MTCT rate increases significantly compared to ExFF. In previous studies, the duration of breastfeeding was arbitrarily determined to be ≤3 months, ≤6 months, etc. Among breastfed children in Nagasaki (included in the ATL Prevention Program in Nagasaki), the prevalence of MTCT was lower among children who were breastfed for ≤6 months than that among children who were breastfed for ≥6 months [16]. However, many healthcare providers have recently limited STBF to ≤3 months, partly because of a higher MTCT rate when carrier mothers breastfed for >3 months than when mothers breastfed for ≤3 months, as shown in the study by Hirata et al. [95].

The mechanism of MTCT prevention by STBF may involve the presence of neutralizing antibodies against HTLV-1 transferred from the carrier mother in utero, which may block MTCT for several months after birth [96]. Another mechanism may involve the lower cumulative number of infected cells entering the gastrointestinal tract due to STBF. MTCT prevention by FTBMF is thought to be caused by the destruction of the infected cells in breastmilk by the freezing and thawing process [97].

In Japan, healthcare providers and mothers are speculated to be interested in STBF and FTBMF as nutritional regimens for MTCT prevention because of their potential to prevent HTLV-1 MTCT while taking advantage of the benefits of breastfeeding [98,99] (e.g., reduction in postpartum anxiety in mothers, formation of mother–infant bonding, and biological effects of the components in breastmilk). A recent systematic review reported that breastfeeding duration is associated with postpartum depression. In addition, postpartum depression was shown to be predicted by breastfeeding cessation in several studies [100]. However, the causes and effects of postpartum depression and short breastfeeding duration are unclear. Screening for depression during pregnancy may be useful in evaluating both aspects [100]. Rocha-Filho et al. showed that both symptomatic and asymptomatic patients with HTLV-1 experienced more anxiety and depression than those experienced by uninfected patients [101]. Thus, it is believed that carrier mothers may have similar experiences.

Even with the implementation of a nationwide antenatal HTLV-1 antibody screening program, the choice of postnatal nutritional regimens varied among regions and healthcare providers. Because of concerns that this situation could cause anxiety for carrier mothers and their families, in 2016, the Ministry of Health, Labour and Welfare (MHLW) of Japan recommended ExFF as the first choice among nutritional regimens for MTCT prevention [102], for which clear evidence had been established. However, evidence for the effectiveness of STBF and FTBMF in preventing MTCT is insufficient because of the small sample sizes and/or methodological issues reported in previous studies. Therefore, we conducted a cohort study, systematic review, and meta-analysis to establish evidence for the effectiveness of STBF and FTBMF in preventing MTCT.

#### 4.2.1. Prospective Cohort Study in Japan

We conducted a prospective cohort study on MTCT prevention using nutritional regimens. Carrier pregnant women were recruited from 92 centers in Japan for 3 years beginning in 2012, and MTCT rates with nutritional regimens were evaluated in their children at 3 years of age. Our study was initiated before the recommendation of the MHLW in Japan [102]. The results were as follows: (1) among 313 HTLV-1 carrier mothers, the proportion of mothers that chose STBF (≤3 months), ExFF, FTBMF, and long-term breastfeeding was 55.0%, 35.1%, 6.1%, and 3.8%, respectively; (2) despite the selection of STBF, 18% of mothers continued to breastfeed for 4 months, and 8% of mothers continued to breastfeed for six months; (3) the MTCT rate in children for whom STBF was selected was 2.3% (4/172), and the risk ratio compared to ExFF was not significantly different (0.365, 95% confidence interval (CI): 0.116–1.145); (4) FTBMF was selected in fewer cases, and the MTCT rate was statistically unreliable. Our study suggests that STBF can be a valid option for the prevention of MTCT. However, as it is not always easy to refrain from breastfeeding within 3 months, both mothers and healthcare providers should be aware of this issue while choosing STBF [94]. In addition, more than half of the children born to the recruited carriers dropped out of follow-up, indicating that a low follow-up rate in children born to pregnant carriers was a major flaw in the screening program.

#### 4.2.2. Systematic Review and Meta-Analysis

**Figure 2:**
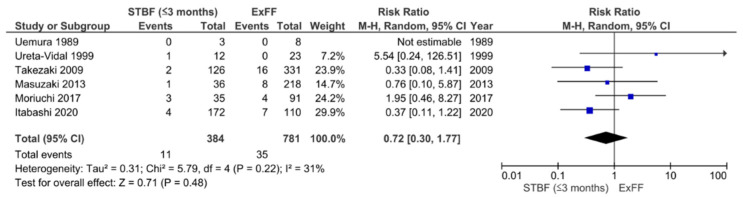
Forest plot of the risk ratios of HTLV-1 MTCT in the ‘STBF ≤3 months’ group compared with that of the ExFF group. STBF, short-term breastfeeding; ExFF, exclusive formula feeding; M–H, Mantel–Haenszel; CI, confidence interval; RR, risk ratio; MTCT, mother-to-child transmission; Events, number of cases with mother-to-child transmission; Total, number of children born to carrier mother; Weight, influence of studies on overall meta-analysis. The figure is reproduced from Miyazawa et al. [112].

**Figure 3:**
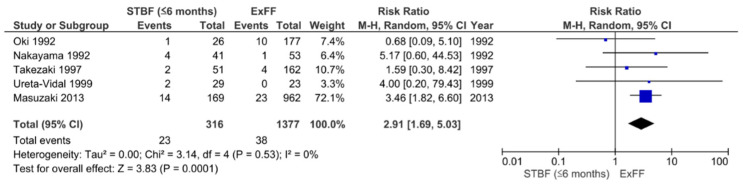
Forest plot of the risk ratios of HTLV-1 MTCT in the ‘STBF ≤6 months’ group compared with that of the ExFF group. STBF, short-term breastfeeding; ExFF, exclusive formula feeding; M–H, Mantel–Haenszel; CI, confidence interval; RR, relative risk; MTCT, mother-to-child transmission; Events, number of cases with mother-to-child transmission; Total, number of children born to carrier mother; Weight, influence of studies on overall meta-analysis. The figure is reproduced from Miyazawa et al. [112].

**Figure 4:**
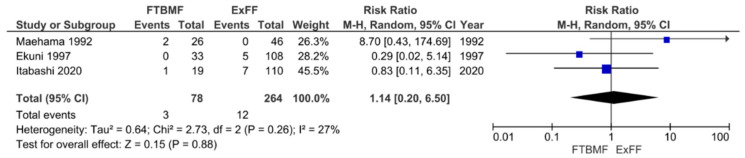
Forest plot of the risk ratios of HTLV-1 MTCT in the FTBMF group compared with that of the ExFF group. FTBMF, frozen-thawed breastmilk feeding; ExFF, exclusive formula feeding; M–H, Mantel–Haenszel; CI, confidence interval; RR, relative risk; MTCT, mother-to-child transmission; Events, number of cases with mother-to-child transmission; Total, number of children born to carrier mother; Weight, influence of studies on overall meta-analysis. The figure is reproduced from Miyazawa et al. [112].

In previous reports, there has been insufficient evidence on the effectiveness of STBF and FTBMF in preventing MTCT compared to ExFF because of the small number of subjects. Therefore, we conducted a systematic review and meta-analysis that incorporated both English and Japanese reports. The definition of STBF varies across articles. In the present study, we defined STBF as breastfeeding for no more than 3 months (STBF ≤ 3 months) or 6 months of life (STBF ≤ 6 months). MTCT was confirmed by the detection of HTLV-1 antibodies in infants after 12 months of life. Finally, 11 articles (i.e., 10 previous studies and our prospective cohort study [94]) were included in the meta-analysis. Six articles on the effect of STBF ≤ 3 months [94,103,104,105,106,107], five articles on STBF ≤ 6 months [104,106,108,109,110], and three articles on FTBMF [94,111,112] were included for the systematic review and meta-analysis [113]. The pooled relative risks of STBF ≤ 3 months, STBF ≤ 6 months, and FTBMF compared with ExFF were 0.72 (95% CI: 0.30–1.77; *p* = 0.48), 2.91 (95% CI: 1.69–5.03; *p* = 0.0001), and 1.14 (95% CI: 0.20–6.50; *p* = 0.88), respectively (Figure 2, Figure 3 and Figure 4) [113]. The results suggest that STBF ≤ 3 months does not increase the risk of MTCT compared to ExFF, but the risk of MTCT may increase by approximately threefold if the duration of STBF is up to 6 months. Although the preventive effect of FTBMF is not significantly different from that of ExFF in MTCT, the number of reports and the number of subjects were small, and the results may not be reliable.

#### 4.2.3. Which Nutritional Regimen Is Best for MTCT Prevention Currently?

In our cohort study and meta-analysis, there was no obvious difference in the MTCM rate between ExFF and STBF ≤ 3 months. In addition, data on FTBMF are lacking. Therefore, from the perspective of preventive effects alone, either ExFF or STBF may be acceptable for postnatal MTCT prevention. However, there are some considerations and issues that need to be addressed when choosing between these nutritional approaches (Table 2).

##### Exclusive Infant Formula Feeding (ExFF)

It is less challenging to perform ExFF than it is to perform STBF, but a major drawback of ExFF is that it does not offer the benefits of breastfeeding. In addition, there are issues regarding the risk of postpartum depression due to low self-esteem owing to not being able to breastfeed and anxiety about the development of HTLV-1-associated diseases [25]. Therefore, counseling should be provided as needed.

##### Short-Term Breastfeeding (STBF)

STBF for ≤3 months appears to offer the advantages of breastfeeding over ExFF, in that the carrier mother can feed her own milk even if it is only for a few months. However, it may not always be easy to refrain from breastfeeding after 3 months, as evidenced by the results of our cohort study [94]. This may be due to problems with the weaning technique and psychosocial conflicts of the mother. If a carrier pregnant woman chooses STBF, then she needs the support of midwives and lactation consultants to minimize stress due to refraining from breastfeeding.

##### Frozen–Thawed Breastmilk Feeding (FTBMF)

FTBMF is very time-consuming when performed at home. Even in our cohort study, only 6.1% of carrier pregnant women opted for it [94]. Given the lack of evidence on its efficacy in preventing MTCT, if it is to be performed, a better indication would be for infants admitted to the neonatal intensive care unit.

##### Other Nutritional Regimens

Regardless of its duration, breastfeeding may also be combined with the use of infant formulas. In recent studies, the rate of MTCT of HIV was extremely high, at approximately 20%, compared with normal breastfeeding or infant formula feeding [114]. It is speculated that mixed feeding may cause gastrointestinal mucosal injury or dysbiosis, which may involve changes in intestinal permeability [115]. However, to date, there is no evidence to inform HTLV-1 carrier women with mixed feeding recommendations, and further studies on the effects of mixed feeding on HTLV-1 MTCT are warranted.

Human milk donated to breast milk banks should be screened for maternal HTLV-1 infection [116]. In theory, feeding banked human milk donated by HTLV-1-uninfected mothers could have the same preventive effect as that of ExFF for infants born to HTLV-1 carriers. However, while banked human milk may provide partial health benefits of ordinary breastfeeding to infants and children [117], it may be unlikely to reduce carrier mothers’ anxiety and/or impairment of mother–child bonding.

#### 4.2.4. Factors Associated with HTLV-1 MTCT

The selection of a feeding regimen is an important factor associated with MTCT of HTLV-1, but the involvement of other factors should also be considered.

Plancoulaine et al. conducted a large genetic epidemiological survey in an HTLV-1-endemic population of African origin from French Guiana. They found the presence of a dominant major gene that predisposes to HTLV-I infection, in addition to the expected familial correlations (mother–offspring and spouse–spouse) due to the virus transmission routes. The authors concluded that this gene appears to account for most infections occurring in children through breastfeeding and can explain, at least in part, the reason why MTCT of HTLV-I only occurs in a certain proportion of children fed by infected mothers [118,119]. A following study by the same group identified a major locus conferring a predisposition to childhood HTLV-1 infection on chromosome 6q27 [120].

An immunological issue that has long been of interest is whether transfer antibodies or antibodies contained in breastmilk play an effective role in preventing mother-to-child infection. Pregnant women infected with HTLV-1 have significantly increased levels of anti-HTLV-1 antibodies, although PVL does not change during pregnancy [121]. This is consistent with the hypothesis that more antibodies are transmitted through the placenta during pregnancy, which may protect against infection in the fetus and early postnatal infants. Rosadas et al. measured anti-HTLV-1/2 IgG antibodies and PVL in paired blood and breastmilk samples from HTLV-1/2-positive mothers and reported that the HTLV-1 PVL and IgG binding ratio were similar in plasma and breastmilk, but that the titer of anti-HTLV-1/2 IgG antibodies in plasma was about 1000 times higher than that in breastmilk [122]. After delivery, HTLV PVL increases in the blood of the mother [123]. Considering the low levels of antibodies in breast milk in addition to the pre- and post-partum trends of PVL and antibodies in infected mothers, it is believed that the preventive effect of STBF on MTCT may not involve IgG antibodies in breastmilk. One of the reasons for the increased risk of MTCT with prolonged breastfeeding may be related to the decrease in transfer antibodies during infancy and the increase in the cumulative intake of infected cells ingested through breastmilk. High maternal PVL has been cited as a risk factor for MTCT [31,124]. This is also reflected in an increase in maternal antibody titer [104].

Substances contained in breastmilk, such as tumor growth factor-β and lactoferrin, which are rich in colostrum [125,126], promote HTLV-I replication [127,128]. Furthermore, lactoferrin expression has been shown to be upregulated during HTLV-1 infection [129]. However, since the respective levels of these components are not constant during lactation and vary from person to person, it is unclear how they actually affect MTCT.

## 5. Conclusions

Since the discovery of HTLV-1 almost 40 years ago, much has been learned about the associated disease and its pathogenesis. MTCT of HTLV-1 became evident within a short time after its discovery, and epidemiological studies and animal studies have shown that prolonged breastfeeding is an important risk factor for MTCT. However, symptoms are rarer in infected infants and children than in adults, and for this reason, there have been no vigorous studies of MTCT, and the detailed mechanisms underlying MTCT remain unknown. Our recent cohort studies and meta-analyses have shown that STBF ≤ 3 months is not significantly different from ExFF in preventing MTCT, which may provide reassurances that STBF can be successfully implemented. However, STBF and ExFF may not be optimal interventions for carrier pregnant women and their children. Breastmilk is the best source of nutrition for infants, and any approach to preventing MTCT by avoiding or limiting breastfeeding must be balanced against the impact on the child’s health and mother–child bonding. In the absence of commercially available vaccines, antivirals, and neutralizing antibodies, to minimize the need for nutritional interventions, it is necessary to identify factors that predispose children born to carrier mothers to MTCT and to thereby predict the development of MTCT with a high degree of accuracy. In addition, further research is needed on the mechanisms underlying prenatal MTCT and its prevention.

## Figures and Tables

**Table 1:** Interpretation of test results for pregnant women.

	Positive	Negative
Primary screening test	Pregnant woman cannot be confirmed as infected: a confirmatory test must be conducted.	Uninfected
Confirmatory test	Infected	Uninfected
PCR (To be performed when the confirmation test is indeterminate)	Infected	Probably uninfected

PCR, polymerase chain reaction.

**Table 2:** Evidence-based selection of nutritional regimens for MTCT prevention.

Nutritional Regimens	Effectiveness on MTCT Prevention	Comments
Exclusive infant formula feeding (ExFF)	Widely used and well evaluated to block MTCT through breastmilk	Approximately 95% or more MTCT prevention No benefits from breastfeeding Concerns about increased risk of postpartum depression and impaired mother–child bonding
Short-term breastfeeding (≤3 months)	No apparent difference in the MTCT prevention effect (vs. ExFF)	Acquisition of some benefits of breastfeeding Approximately 18% of children exceed 4 months of breastfeeding Need to provide adequate support for weaning No data on the preventive effect of postpartum depression or impairment of mother–child bonding
Short-term breastfeeding (≤6 months)	Approximately three times increased risk of MTCT (vs. ExFF)	Better to avoid this regimen
Frozen–thawed breastmilk feeding	Unknown effectiveness of MTCT prevention due to lack of sufficient case accumulation (vs. ExFF)	Time-consuming Considered for use in infants admitted in the NICU No data on the preventive effect of postpartum depression or impairment of mother–child bonding
Mixed feeding	Unknown effectiveness of MTCT prevention due to lack of data (vs. ExFF)	Concerns about increased risk of MTCT due to damage to the intestinal mucosa Better to avoid this regimen
Banked human milk	No data available, but expected to be as effective as ExFF in preventing MTCT	No use of breast milk from untested HTLV-1 donors No data on the preventive effect of postpartum depression or impairment of mother–child bonding

Note: It should be noted that ~5% of prenatal infections cannot be avoided regardless of which nutritional regimen is chosen. MTCT, mother-to-child transmission; NICU, neonatal intensive care unit.
